# MiR-195-5p and miR-205-5p in extracellular vesicles isolated from diabetic foot ulcer wound fluid decrease angiogenesis by inhibiting VEGFA expression

**DOI:** 10.18632/aging.203393

**Published:** 2021-08-09

**Authors:** Jing Liu, Jiahuan Wang, Wan Fu, Xiaoyi Wang, Hongxing Chen, Xiaoying Wu, Guojuan Lao, Yuxi Wu, Mengdie Hu, Chuan Yang, Li Yan, Meng Ren

**Affiliations:** 1 Department of Endocrinology, Sun Yat-Sen Memorial Hospital, Sun Yat-Sen University, Guangzhou 510120, China

**Keywords:** extracellular vesicles, diabetic foot ulcers, microRNAs, angiogenesis, wound fluid

## Abstract

Diabetic foot ulcers are recalcitrant to healing, and poor angiogenesis is considered as the main contributing factor. We aimed to explore the effect of extracellular vesicles (EVs) derived from wound fluids on new vessel formation in diabetic foot ulcers. EVs were isolated from wound fluids of diabetic foot ulcers (DF-EVs). The inhibitory effect of DF-EVs on human umbilical vein endothelial cells (HUVECs) and wound healing was tested. To elucidate the potential mechanism of these effects, we screened the differentially expressed microRNAs (miRNAs) in DF-EVs via microarray analysis and verified the upregulation of miR-195-5p and miR-205-5p in DF-EVs via quantitative real-time polymerase chain reaction (qRT-PCR). Further dual-luciferase reporter assays and overexpression experiments proved these two miRNAs inhibited the expression of vascular endothelial growth factor A (VEGFA) directly to the 3′ untranslated region (UTR) of VEGFA and, in turn, promoted an inhibitory effect of DF-EVs on angiogenesis and wound healing in patients with diabetic foot ulcers. Our study shows EVs in the wound fluids of diabetic foot ulcer lesions carrying antiangiogenic miR-195-5p and miR-205-5p negatively regulated angiogenesis and wound healing in patients with diabetic foot.

## INTRODUCTION

Wound healing is a complex and ordered process including four overlapping phases of coagulation, inflammation, proliferation and remodeling [1] during which new vessel formation is key to provide oxygen and nutrients, facilitating the removal of debris and promoting wound healing [2]. However, the normal progression of wound healing is disrupted in patients with diabetes. Fewer capillaries and decreased lumen area in diabetic wounds are observed *in vivo* [3], and cellular experiments have validated that both advanced glycation end products (AGEs) and high glucose impaired angiogenesis [4], the mechanism of which remains unclear.

Extracellular vesicles (EVs) are cell-derived microparticles in the extracellular medium that have a lipid bilayer and can be found in various body fluids. Harboring original cell-derived specific cargoes, including functional nucleic acids (mRNA, miRNA or other RNA species) and proteins, EVs can alter the phenotype and affect the function of target cells [5]. Wound fluid constitutes the microenvironment of repairable skin cells and wound healing and contains exudate from the bloodstream, activated local cells and migrated cells [6]. Although the important role of EVs in skin have been reported, studies on the role of DF-EVs are in their infancy.

MiRNAs are small noncoding RNAs of 21–22 nucleotides that mediate post-transcriptional gene silencing and subsequently repress protein synthesis by incorporating into the RNA-induced silencing complex (RISC). Numerous studies have demonstrated that diabetes disrupts the expression of miRNAs, which are involved in diabetic micro- and macrovascular complications development [7, 8]. Storage in EVs protects miRNAs from degradation, and miRNAs can be transferred to their recipient cells by EVs, which facilitates the information transmit and exchange. The regulatory role of miRNAs at the post-transcriptional level has been studied most often in regards to EV contents. Research has shown that exosomes isolated from pericardial fluids improve therapeutic angiogenesis partially by transferring cardiovascular miRNAs [9], indicating that EVs in body fluids can effectively regulate the formation of new blood vessels.

Thus, in current study, we profiled the miRNAs in EVs derived from wound fluids of diabetic foot ulcers (DF-EVs) and nondiabetic wounds (Control-EVs) and found that miR-195-5p and miR-205-5p, which can regulate VEGFA expression, were upregulated in DFs. Furthermore, we explored the effect and mechanism of DF-EVs on angiogenesis and wound healing.

## RESULTS

### Characterization of isolated EVs

**Figure 1:**
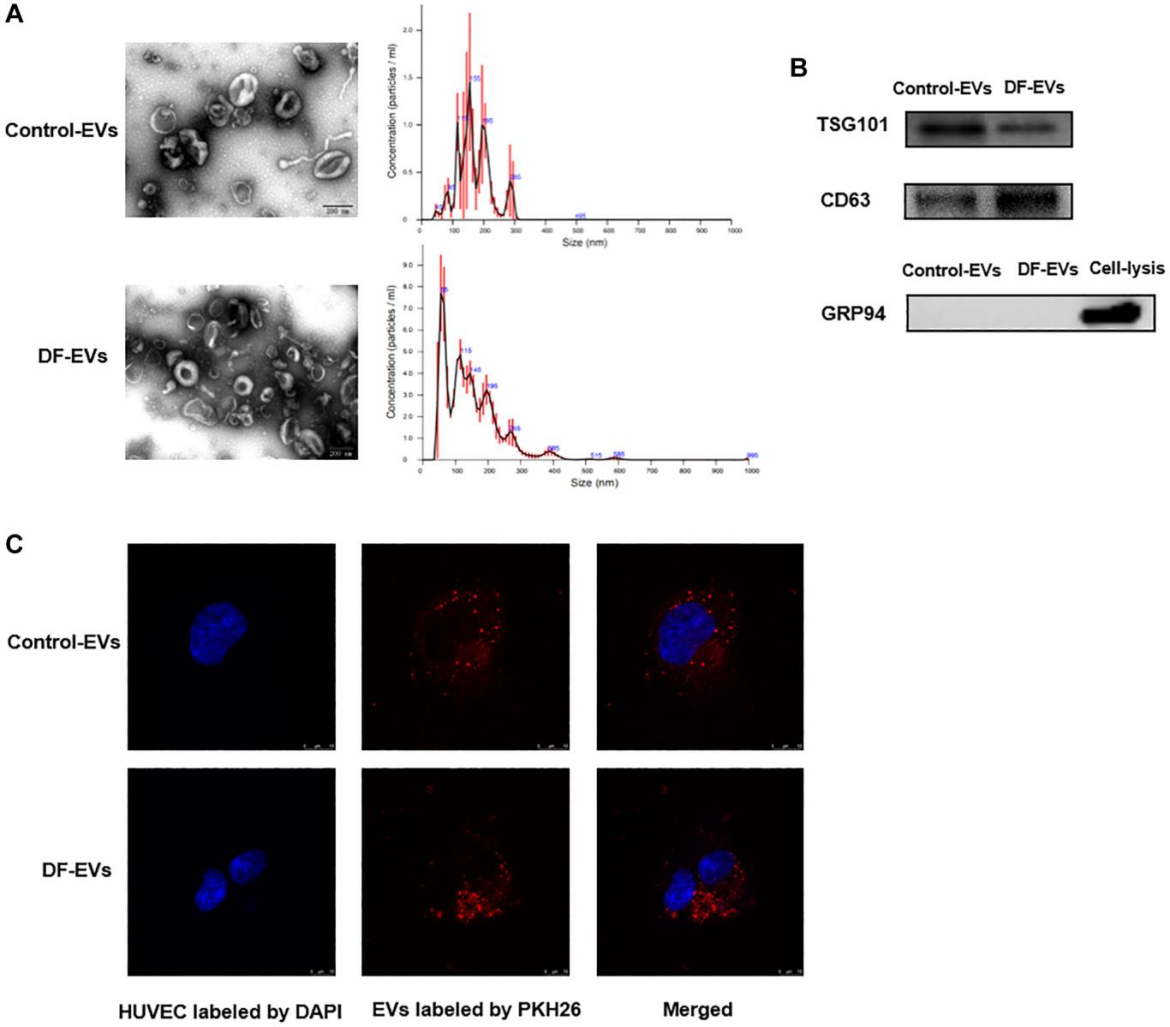
**Characteristics of EVs derived from different wound fluids.** (**A**) TEM analysis and NTA analysis of EVs. Scale bar: 200 nm. (**B**) EVs-related markers were detected by Western blotting. (**C**) EVs were labeled with PKH26 and co-incubated with HUVECs, and the representative images photographed by confocal microscope are showed above. Scale bar: 10 μm. At least three replicates of each experiment were performed.

Although EVs from chronic wound fluids were purified in a previous study using an exosomal isolation kit [10], these EVs have not been identified or characterized. Therefore, DF-EVs isolated in this study were first characterized according to the International Society of Extracellular Vesicles guidelines [11]. The transmission electron microscopy (TEM) image shows that EVs isolated by differential centrifugation were intact, spherical vesicles with a diameter of approximately 50–100 nm (Figure 1A). NTA showed that most of the EVs ranged in size from 50–150 nm, with a peak at approximately 55 nm (Figure 1A). Western blot analysis indicated that the EV marker proteins TSG101 and CD63 were strongly enriched in the isolated EVs, and the endoplasmic reticulum protein GRP94 was negative (Figure 1B). To study the internalization of EVs by HUVECs, the purified EVs were fluorescently labeled with PKH26. After incubation with the labeled EVs, confocal microscopy revealed a punctate pattern of red fluorescence in the cytoplasm of HUVECs, counterstained for nuclei with blue fluorescent DAPI (Figure 1C).

### DF-EVs inhibited HUVEC angiogenesis *in vitro* and *in vivo*

**Figure 2:**
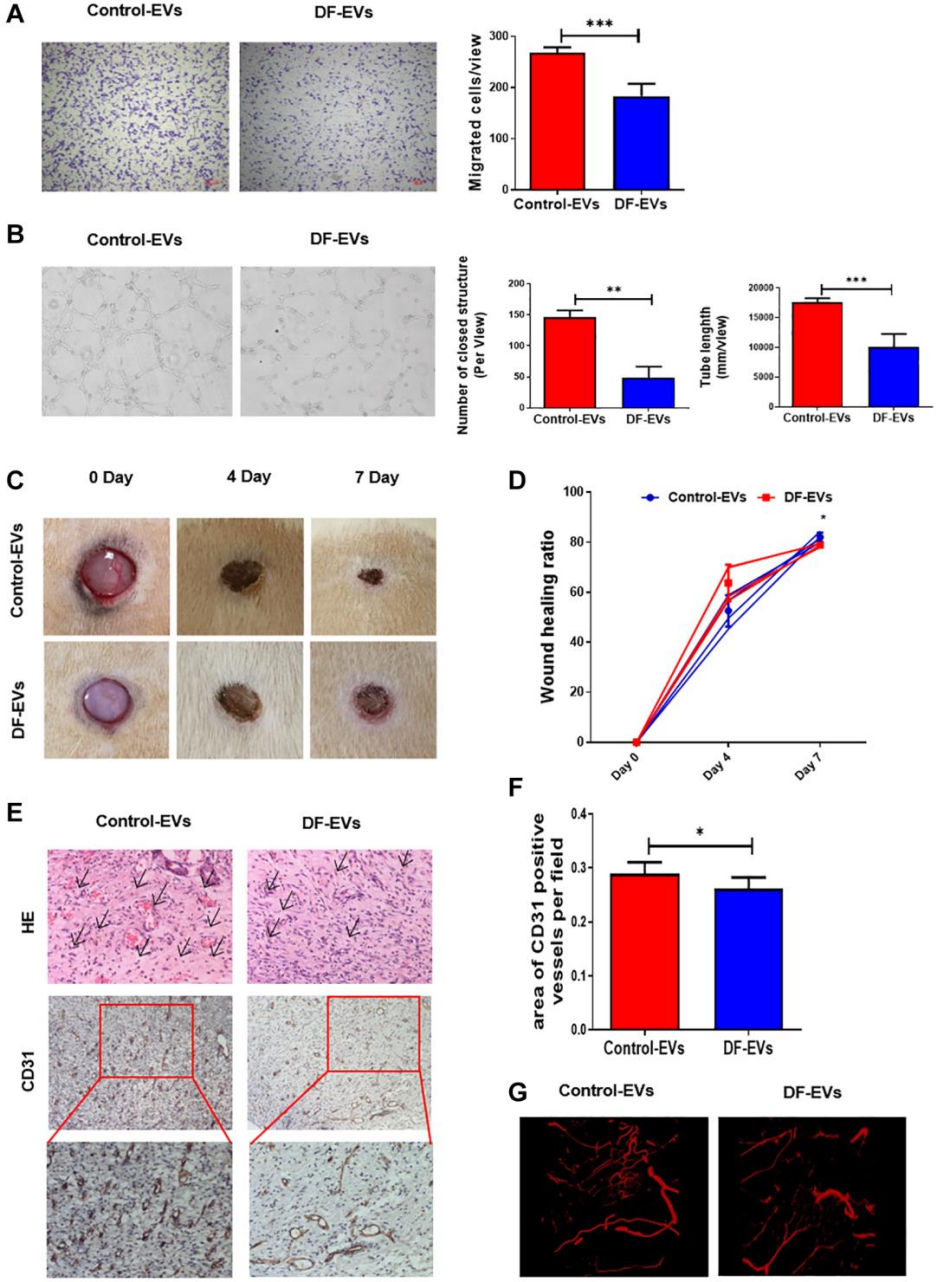
**DF-EVs inhibited HUVEC angiogenesis *in vitro* and *in vivo*.** (**A**) Transwell assay of HUVECs treated with 5 μg/ml DF-EVs or 5 μg/ml Control-EVs. Scale bar: 100 μm. (**B**) *In vitro* tube formation assay of HUVECs treated with 5 μg/ml DF-EVs or 5 μg/ml Control-EVs. (**C**, **D**) Representative images of cutaneous wounds with 200 μg Control-EVs and 200 μg DF-EVs treatment at day 0, 4 and 7 post-wounding. (**E**, **F**) HE and CD31 staining of wound section with different treatments at day 7 post-wounding. (**G**) Microangiography analysis of local wound treated with DF-EVs or Control-EVs. ^*^P < 0.05, ^***^P < 0.001. At least three replicates of each experiment were performed.

To reveal more details about the effect of cellular DF-EV uptake, HUVECs were treated with 5 μg/ml DF-EVs or Control-EVs, and their migration and vessel formation capacity were analyzed. HUVEC proliferation was assessed using the cell counting kit-8 (CCK-8) assay, revealing that 5 μg/ml DF-EVs had no effect on cell proliferation (Supplementary Figure 1A). Furthermore, transwell assay indicated that, compared with the control group, HUVECs treated with DF-EVs showed an obviously decreased cell migration capacity (Figure 2A). At the same time, we evaluated the vessel formation capacity using an *in vitro* tube formation assay. As shown in Figure 2B, considerably less vessel formation, including a reduced number of closed structures and shorter tube length, was observed in HUVECs cocultured with DF-EVs compared with the control group.

We then explored the effect of DF-EVs on the cutaneous wound healing in rats. Administration of DF-EVs slowed the wound healing process, as the relatively larger wound area was seen at day 7 post-wounding compared to the control group (Figure 2C and 2D). Consistent with the results of HUVEC tube formation analysis, the wounds treated with DF-EVs exhibited less extensive new blood vessel formation in the local granulation tissue than the control group on day 7 according to general HE and immunohistochemical staining (Figure 2E and 2F). At the same time, local wound microangiography analysis revealed obviously fewer microvessels in the group treated with DF-EVs (Figure 2G). These *in vivo* data collectively suggested that DF-EVs delayed wound healing by impairing angiogenesis, indicating a possible role of DF-EVs in modulating the angiogenic capacity of endothelial cells (ECs) in DF ulcers.

### MiR-195-5p and miR-205-5p were upregulated in DF-EVs

**Figure 3:**
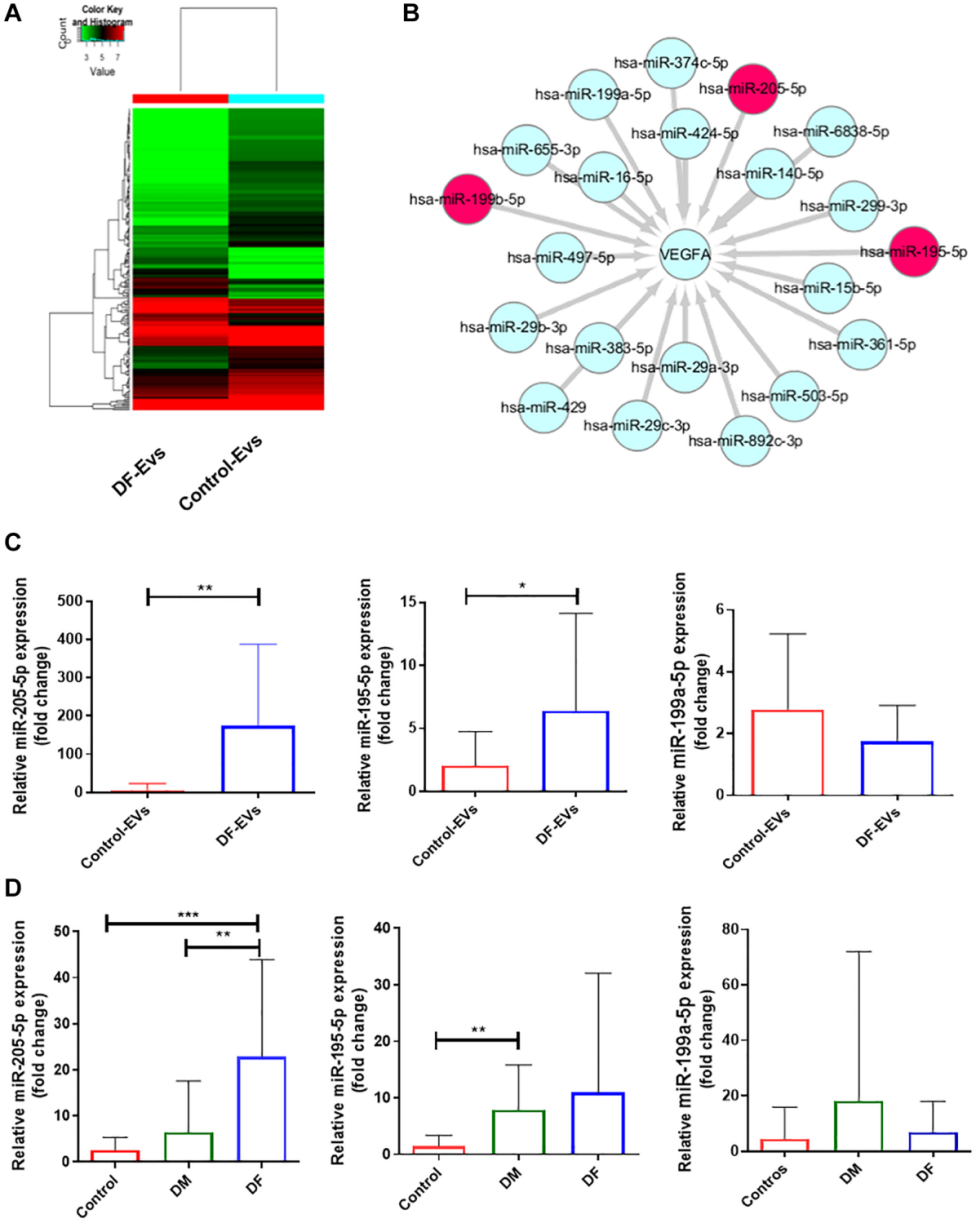
**MiRNA expression of DF-EVs.** (**A**) Differential expression levels of miRNAs between Control-EVs and DF-EVs were presented in a heatmap. (**B**) MiRNA network predicted to target VEGFA using FunRich software. (**C**) Expression levels of candidate miRNAs in DF-EVs were quantitated by qRT-PCR. (**D**) Candidate miRNAs were detected in sera of nondiabetic patients and diabetic patients with or without foot ulcers. ^*^P < 0.05, ^**^P < 0.01, ^***^P < 0.001. At least three replicates of each experiment were performed.

To explore the functional cargo of DF-EVs, we isolated RNA from DF-EVs and Control-EVs and conducted microarray-based miRNA profiling. In total, 211 differentially expressed miRNAs were identified, including 58 that were upregulated and 153 that were downregulated in DF-EVs compared to Control-EVs (Figure 3A). Because VEGFA is a well-known signaling protein involved in vessel formation, we constructed a network encompassing the miRNAs that are likely to target and regulate the expression of VEGFA using FunRich software. In the network (Figure 3B), miR-195-5p, miR-199b-5p and miR-205-5p were differentially expressed in the DF-EVs as determined in the microarray assay. Based on this network, these 3 miRNAs were selected for further study. First, to confirm the reliability of the miRNA expression, an equal amount of DF-EVs and Control-EVs was subject to RNA extraction, the level of miRNA expression was detected by qRT-PCR. The expression level of miR-205-5p in DF-EVs was more than 100-fold higher than that in Control-EVs, and miR-195-5p expression was also significantly higher as shown in Figure 3C. However, there was no difference in the level of miR-199b-5p. A similar result was also obtained from EVs derived from the sera of patients with diabetes mellitus (DM) without or with DF, and nondiabetic patients (Control). The qRT-PCR results showed that the miR-205-5p level in serum-derived EVs from DF patients was significantly higher than those in EVs derived from DM and control group sera (Figure 3D). However, the serum levels of miR-195-5p and miR-199b-5p in the DF group were not different from those in the other two groups, indicating that the EVs with upregulated miR-195-5p expression in the local wound fluid were potentially not derived from peripheral circulation.

### Overexpression of miR-195-5p and miR-205-5p inhibited angiogenesis

**Figure 4:**
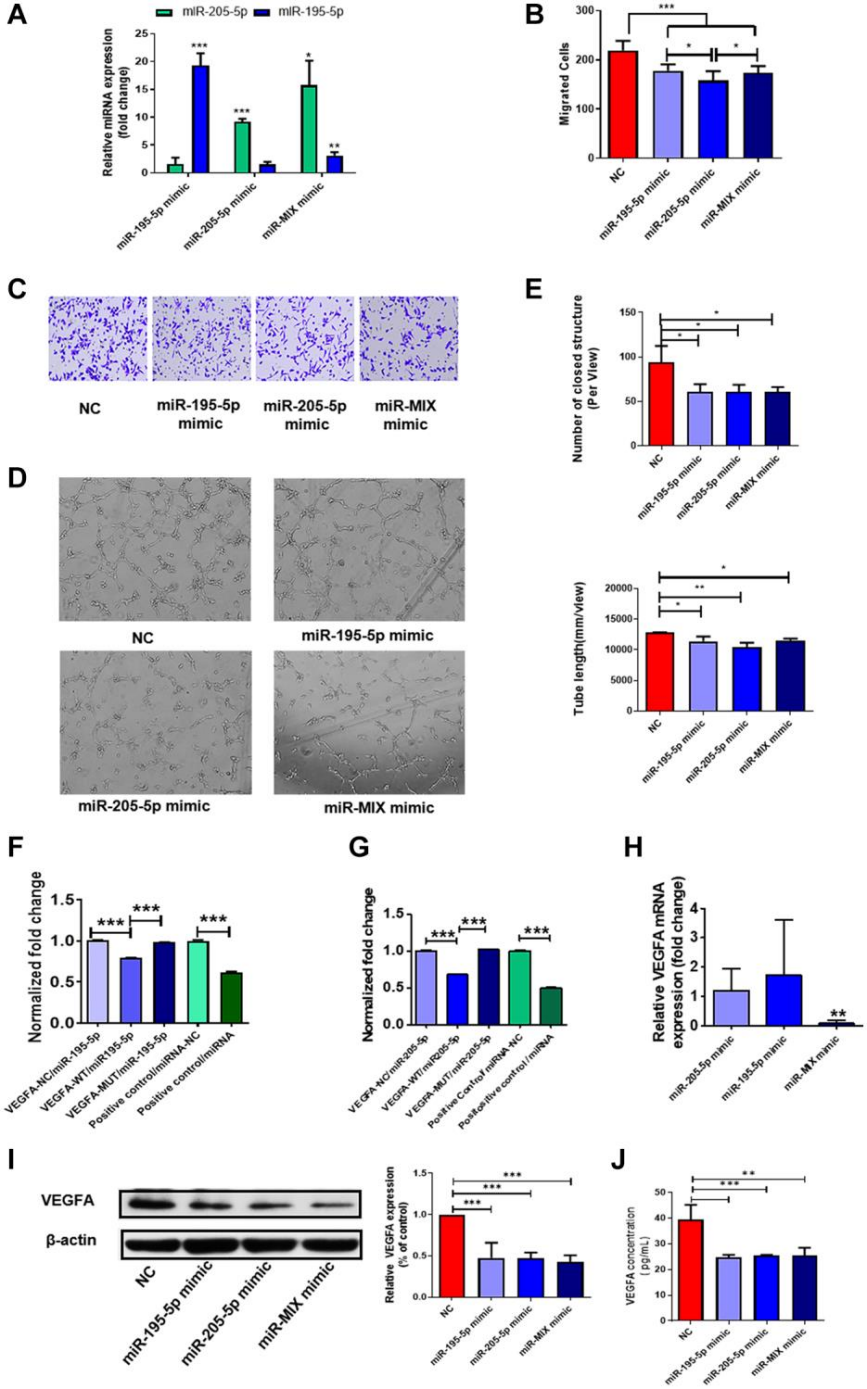
**MiR-195-5p and miR-205-5p inhibited angiogenesis through reducing VEGFA.** (**A**) MiR-195-5p and miR-205-5p expression level in HUVECs transfected with miRNA mimics was determined by qRT-PCR. (**B**, **C**) Transwell assay of HUVECs transfected with either or both of miR-195-5p and miR-205-5p mimics. (**D**, **E**) *In vitro* tube formation assay of HUVECs transfected with either or both of miR-195-5p and miR-205-5p mimics. (**F**, **G**) VEGFA-3′-UTR luciferase reporter assays of HUVECs in the presence of miR-195-5p and miR-205-5p mimics separately. (**H**, **I**) The expression level of VEGFA in HUVECs overexpressing either or both of the miRNA mimics were detected by qRT-PCR and western blot separately. (**J**) The concentration of VEGFA in culture medium of HUVECs overexpressing either or both of the miRNA mimics were detected by Elisa. ^*^P < 0.05, ^**^P < 0.01, ^***^P < 0.001. At least three replicates of each experiment were performed.

Collectively, the data indicated that DF-EVs may transfer miR-195-5p and miR-205-5p into HUVECs and inhibit angiogenesis by targeting VEGFA. Thus, to verify whether DF-EVs exert antiangiogenic effects on HUVECs by transporting these two miRNAs, we confirmed the transfection efficiency of miRNA mimics (Figure 4A) and evaluated the migration and vessel formation capacities of HUVECs overexpressing these two miRNAs. HUVECs migrated slower when either or both of these two miRNAs were overexpressed compared to the negative control (Figure 4B and 4C). Furthermore, overexpression of either or both miRNAs strongly suppressed the tube formation capacity of HUVECs (Figure 4D), and statistical results showed that the number of closed structures and tube length were both significantly less than those in the scrambled sequences (negative control, N.C.) group (Figure 4E).

To further confirm that these two miRNAs modulate angiogenesis by directly targeting the 3′-UTR of VEGFA, we performed 3′-UTR luciferase reporter assays. Compared with the control, the luciferase activity of the VEGFA-wild type (WT) 3′-UTR reporter dropped to 80% and 68% after transfection with miR-195-5p and miR-205-5p mimics, respectively, as shown in Figure 4F and 4G. Mutation of the predicted target sites completely abolished the repressive effect of miR-195-5p or miR-205-5p mimics on reporter gene expression (Figure 4F and 4G), demonstrating that miR-195-5p and miR-205-5p directly target VEGFA. Consequently, we concluded that these two miRNAs regulate the expression of VEGFA by directly binding to the 3′-UTR. Moreover, to verify this effect in ECs, we explored the expression of VEGFA at the mRNA and protein levels in HUVECs overexpressing these two miRNAs. Notably, the expression of VEGFA mRNA was decreased in HUVECs upregulating both miRNAs, while there was no difference in HUVECs overexpressing miR-195-5p or miR-205-5p alone (Figure 4H). However, a single miRNA could significantly reduce the expression of VEGFA at the protein level, as the concentration of VEGFA secreted into the culture medium and of VEGFA in the cells decreased significantly (Figure 4I and 4J). However, simultaneous overexpression of both miRNAs did not further repress VEGFA expression inside or outside HUVECs.

### MiR-195-5p and miR-205-5p transferred by DF-EVs regulate VEGFA expression

**Figure 5:**
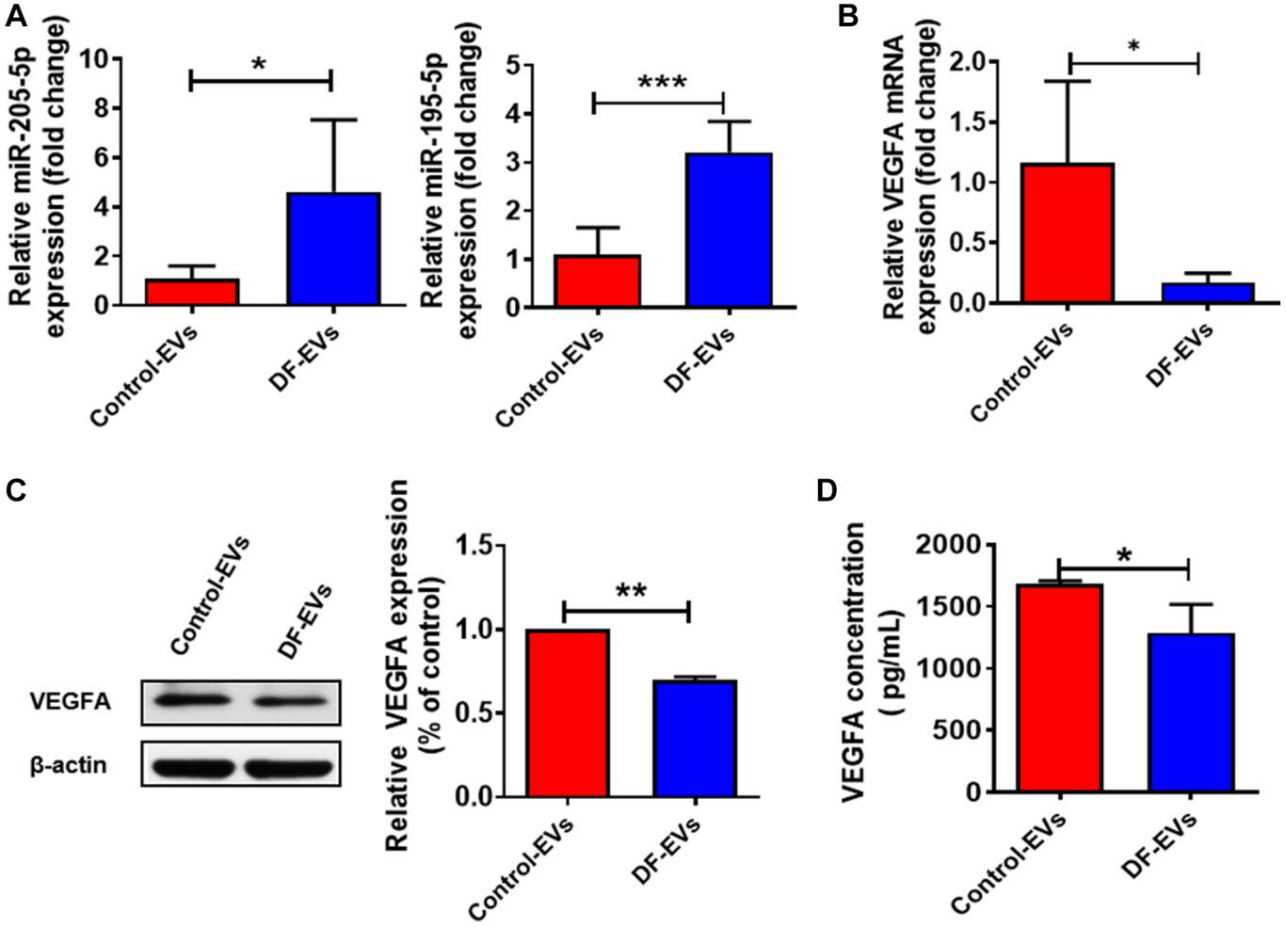
**DF-EVs regulate VEGFA expression through miR-195-5p and miR-205-5p.** (**A**) Expression level of MiR-195-5p and miR-205-5p in HUVECs treated with DF-EVs or Control-EVs. (**B**, **C**) Expression level of VEGFA in HUVECs treated with DF-EVs or Control-EVs were detected by qRT-PCR and western blot separately. (**D**) The secretion of VEGFA in culture medium of HUVECs treated with DF-EVs or Control-EVs were detected by Elisa. ^*^P < 0.05, ^**^P < 0.01.

*In vitro* data presented above collectively suggested that miR-195-5p and miR-205-5p from DF-EVs may impair wound healing in DF ulcers by inhibiting angiogenesis. Since DF-EVs functioned by transferring functional contents into recipient cells, we detected the expression levels of miRNAs in HUVECs treated with DF-EVs or Control-EVs. The qRT-PCR results suggested that the levels of both miR-195-5p and miR-205-5p (Figure 5A) were significantly increased by more than 3-fold in the DF-EVs treatment group. Moreover, the mRNA expression level of VEGFA was also significantly decreased in the DF-EVs group (Figure 5B). Consistently, the protein level of VEGFA was also reduced in HUVECs treated with DF-EVs (Figure 5C). Because VEGFA is a secretory protein and functions in the extracellular space, we tested the concentration of secreted VEGFA in the cell culture medium by ELISA, which indicated that the secretion of VEGFA was significantly reduced in the DF-EVs group (Figure 5D).

In addition, we also investigated whether other target genes are potentially controlled by these two miRNAs in DF. A recent study found that the upregulation of YAP1 plays a critical role in promoting angiogenesis during the development of diabetic retinopathy. Furthermore, ZEB1 was also found to be a key regulator of diabetic cutaneous wound healing [12]. Considering that YAP1 and ZEB1 are predicted to be target genes of both miR-195-5p and miR-205-5p, we also examined their expression levels in HUVECs treated with DF-EVs or overexpressing both or either of the above mentioned miRNAs. However, the mRNA levels did not change significantly in HUVECs cocultured with DF-EVs or overexpressing miR-195-5p and/or miR-205-5p (Supplementary Figure 2). Therefore, EVs with enhanced miR-195-5p and miR-205-5p expression in the wound fluid may be transferred into HUVECs and preferentially target VEGFA to inhibit VEGFA, thus playing an antiangiogenic role.

## DISCUSSION

In the current study, we identified EVs derived from the wound fluid of diabetic foot ulcers and confirmed their inhibitory effect on angiogenesis *in vivo* and *in vitro*. We observed that DF-EVs significantly inhibited the migration and vessel formation capacity of HUVECs, resulting in decreased angiogenesis. This effect can be explained by the enhanced miR-195-5p and miR-205-5p expression in DF-EVs, which were transferred to ECs. Here, we reported that miR-195-5p and miR-205-5p in EVs inhibited angiogenesis by targeting VEGFA, which is the most potent angiogenic molecule. Furthermore, in addition to the antiangiogenic miR-195-5p and miR-205-5p, DF-EVs also contain other miRNAs with anti- or proangiogenic functions. The antiangiogenic response to DF-EVs may be caused by the overall antiangiogenic miRNAs transferred by the same vesicle, and we herein found two antiangiogenic miRNAs incorporated into ECs and targeting an identical gene. Furthermore, we should not ignore the possibility that EVs enriched in proangiogenic miRNAs may be less inclined to be taken up by ECs or more likely to be degraded in recipient cells under diabetic conditions.

In the microenvironment of wound healing cells, the wound fluid content significantly influences the reconstruction of the extracellular matrix and the healing activity of skin cells [13]. Studies have also found high concentrations of inflammatory cytokines in nonhealing wound fluid, thus leading to poor mitogenic activity of fibroblasts and poor wound healing [14]. A recent study also found that miR-21 was enriched in exosomes from chronic wound fluid, which may originate from keratinocytes and be transferred into macrophages in local wounds to mediate their conversion. In our current research, we discovered the inhibitory effect of DF-EVs on wound healing by transferring antiangiogenic miRNAs into ECs, which suggests that EVs in biological fluids play a nonnegligible role in intercellular communication and can potentially promote disease progression. Previous studies have confirmed that EVs from keratinocytes can regulate gene expression in dermal fibroblasts and thereby promote tissue repair and wound healing [15]. Our previous study showed that EVs derived from HUVECs in the context of diabetes could inhibit collagen synthesis in skin fibroblasts and thus delay wound healing in diabetic ulcers [16]. Moreover, EVs from normal fibroblasts were able to significantly accelerate wound healing in db/db mice, a genetically diabetic mouse model, by improving angiogenesis as well as the migration and proliferation of keratinocytes [17]. Taken together, these findings indicated that the functional EVs from wound healing cells may use the wound fluid as a medium to transfer EVs and miRNA messages to other cells, mediating intercellular communication. Taken as a reflection of the clinical wound condition, wound fluids are complex exudates with a variety of endogenous and exogeneous constituents derived from peripheral circulation, local cells or infected bacteria [6]. However, while identifying the origin of EVs in wound fluids is challenging, elucidating the functional contents impairing wound healing remains a feasible strategy to develop novel targeted therapies.

We therefore aimed to explore the antiangiogenic cargoes from EVs of diabetic wound fluids and confirmed the presence of two miRNAs that potentially lead to EC dysfunction and impaired angiogenesis. MiR-205-5p is a known antiangiogenic factor, and its role in the development of several cancers has been extensively studied. Reduction in miR-205-5p expression and subsequent inadequate suppression of VEGFA lead to excessive activation of VEGF signaling and neovascularization in hepatocellular carcinoma [18] and bladder cancer [19], among others, and these events are critical for tumor growth and spread. In addition, oxidative stress suppressed the expression of miR-205-5p in retinal epithelial ARPE-19 cells; thus, VEGFA expression and angiogenesis increased, which may be a potential mechanism of diabetic retinopathy [20]. In our study, we found enhanced miR-205-5p expression in EVs of diabetic wound fluids, and administration of EVs containing miR-205-5p significantly inhibited angiogenesis in wounds of normal mice. Although it has been revealed that miR-205-5p suppresses VEGFA expression at the post-transcriptional level in mesenchymal stem cells (MSCs), inhibition of which can significantly improve the therapeutic effect of MSCs on diabetic wounds by further promoting vascularization [21], this is the first study to identify the increased level of this antiangiogenic miRNA in the local wound of DF ulcers encapsulated in EVs, which could effectively protect cargo miRNAs from degradation and transfer into recipient cells. Moreover, miR-195-5p was found to be upregulated in the circulation of patients with pre-eclampsia [22], thereby leading to the suppression of VEGFA and an antiangiogenic status. In the context of DM, high glucose treatment enhanced the level of miR-195-5p expression in cardiomyocytes and was implicated in the development of diabetic cardiomyopathy [23]. Thus, both miRNAs are known to be closely related to blood vessel formation and may play a potential role in the pathology of DM. In the current study, for the first time, upregulated miR-195-5p and 205-5p expression was found concurrently in DF-EVs, and these miRNAs were shown to be transmitted into ECs targeting the same genes, although they seemed to have no synergistic effect. Our data showed that VEGFA mRNA and protein levels in HUVECs integrated with DF-EVs or overexpressing both miRNAs were significantly decreased. However, single miRNA upregulation in HUVECs did not suppress the mRNA expression of VEGFA but inhibited the protein level of VEGFA, which indicated that single overexpression of miR-205-5p or miR-195-5p inhibited VEGFA expression at the post-transcriptional level. However, simultaneously overexpressing both miRNAs did not further inhibit VEGFA expression at the transcriptional level and had a reinforced effect. We also identified that these two miRNAs functioned in diabetic wounds through novel intercellular communication via EVs.

VEGFA is a member of the VEGF family, whose signaling is often considered the critical step in physiological angiogenesis. Moreover, VEGFA functions to maintain angiogenic stimuli and is involved in wound repair. However, the mechanism of decreased VEGFA expression and impaired angiogenesis in diabetic skin lesions is still unclear. VEGFA expression is regulated by multiple mechanisms at multiple levels in physiological and pathological processes [24]. Although hypoxia has been considered the strongest inducer of VEGFA transcription [25], epigenetic regulation has recently drawn increasing attention, and the role of miRNA has been confirmed in multiple studies [26, 27]. In line with previous studies, our data revealed that elevated miR-195-5p and miR-205-5p levels in EVs regulate the expression of VEGFA by directly binding to the 3′-UTR region of VEGFA. It is suggested that the inhibitory effect on HUVECs may be mediated through the inadequate activation of VEGFA signaling due to the repression of VEGFA. Considering that these two miRNAs may function by targeting other genes, we also investigated other known genes related to wound healing and angiogenesis in recipient cells. However, the results were negative, which indicated that these miRNAs may specifically bind the 3′-UTR of VEGFA and exert an inhibitory effect in ECs.

EVs are involved in the development of various diseases, including diabetes and its complications, and their quantity and cargoes in biological fluids vary under different pathological and physiological conditions. EVs act on recipient cells by activating cellular surface receptors and signaling pathways and becoming internalized or fused with target cells, and their cargoes delivered into target cells also differ in fate and function [28]. Throughout the process of cell communication, the specific cargoes (including miRNAs and proteins), biogenesis and release, and targeting of the recipient cells of EVs have been poorly studied; however, these processes may be major, and further investigation is essential. Studies have illustrated that sorting machineries and specific RNA-binding proteins can be modified and mediate RNAs with specific motifs packed into EVs [29, 30]. In our study, increased expression levels of the antiangiogenic miR-195-5p and miR-205-5p were found in diabetic wound fluids, and investigating whether the mechanism of cargo sorting into EVs and EVs release into body fluids are altered under diabetic conditions and whether these changes can be rectified is of particular interest. Thus, given that EVs play a critical role in various diseases, the exact molecular mechanisms implicated in EVs biogenesis and secretion warrant further exploration. Elucidating the fate of miRNAs inside EVs and promoting their degradation in recipient cells may be another feasible way to reverse the adverse effects of DF-EVs.

In summary, our study has uncovered that in the DF ulcer microenvironment, EVs in the wound fluid appear to inhibit angiogenesis and wound healing, which is at least partially mediated by increased expression of antiangiogenic miR-205-5p and miR-195-5p, which in turn suppress the expression of the key proangiogenic factor VEGFA.

## MATERIALS AND METHODS

### Ethics statement

Investigation has been conducted in accordance with the ethical standards, the Declaration of Helsinki and national and international guidelines. The study has been approved by the review board of Sun Yat-Sen Memorial Hospital affiliated with Sun Yat-Sen University.

### Patients and serum sample collection

Demographic information including age, sex, fasting blood glucose (FBG), hemoglobin A1C (HbA1C), serum creatinine (Cr), triglyceride (TG), total cholesterol (TC), high-density lipoprotein cholesterol (HDL-C) and low-density lipoprotein cholesterol (LDL-C) were recorded for all patients with DF, diabetes mellitus (DM) and non-diabetic patients undergoing thyroidectomy (controls) (Supplementary Table 1). Fasting serum samples were obtained from patients with DF, DM and controls. Serum samples were collected between 6:00–8:00 a.m. using separation gel coagulation-promoting blood collection tubes followed by centrifugation at 1000 rpm for 10 min. Then, the serum in the upper layer was immediately transferred to a sterile centrifuge tube and stored at –80°C. EVs was isolated from serum of different patients separately.

### Collection of wound fluid

Wound fluids used for EVs isolation were collected from the ulcer sites of patients with DF, and the postoperative drainage fluid was collected from nondiabetic patients undergone subtotal or total thyroidectomy. DF wound fluid was collected according to a previous study with appropriate modification [31]. Briefly, the ulcers were rinsed with sterile saline and blotted dry with sterile gauze after being subjected to debridement and effective infection control practices. New sterile gauze was then applied to the ulcer surface until completely saturated. The gauze soaked with wound fluid was then moistened with 10 ml of PBS and incubated for 2 h at 4°C. Finally, the wound fluid was squeezed from the gauze into a sterile centrifuge tube on ice with syringe. Drainage fluid was collected from control group subjects on the morning of the first postoperative day by pouring into sterile centrifuge tubes directly from the drainage bag. After collection in the tubes, wound fluid was immediately centrifuged at 300 g for 20 min followed by centrifugation at 3000 g for 20 min. All centrifugations were performed at 4°C. Then, the supernatants were transferred to new sterile containers and frozen at –80°C for future use.

### Isolation of EVs

The EV isolation method varied depending on the subsequent experimental design. EVs in serum was precipitated with an equal volume of polyethylene glycol (PEG) 6000 buffer (16% PEG 6000, 1 mM NaCl) overnight at 4°C, before centrifugation at 12,000 g for 60 min according to a previous study [32]. The EVs pellet was finally dissolved in PBS. For cellular experiments, DF-EVs were isolated by differential ultracentrifugation [33]. Briefly, the fluids were centrifuged at 10,000 g for 30 min, and then the supernatants were centrifuged at 120,000 g (Beckman Coulter Optima XC-100 ultracentrifuge, Fullerton, CA, USA) for 70 min at 4°C. After suspending in PBS, a second ultracentrifugation was needed to precipitate the EVs under the same conditions. The EVs pellet was finally suspended in PBS. The protein concentration of EVs was detected with the Micro BCA Protein Assay Kit (CWBio, Jiangsu, China).

### EVs characterization

Exosome size distribution was analyzed by Nanosight LM 10 (Malvern Instruments Ltd., Malvern, UK). Particle scatter was recorded using nanoparticle tracking analysis (NTA) acquisition and analysis software. The exosomes were visualized and imaged by transmission electron microscopy (JEM-1200EX, JEOL). The EV markers were identified with western blot. Total protein was extracted from exosomes according to the established protocol. Antibodies included rabbit anti-CD63 and rabbit anti-TSG101.

### Cell culture

HUVECs were purchased from American Type Culture Collection (ATCC) and cultured in Endothelial Cell Medium (ECM, ScienCell, San Diego, CA, USA). After reaching 70% confluence, HUVEC were cocultured with 5 μg/ml DF-EVs or Control-EVs (HyClone, Logan, UT, USA) for 24 h and harvested for further assays and analysis of VEGFA and miRNA expression. The HUVEC supernatants were collected to measure VEGFA using a VEGFA ELISA kit (Elabscience, Wuhan, China).

### EVs Labeling with PKH26 and HUVEC uptake

EVs isolated by differential centrifugation were labeled with the PKH26 Red Fluorescent Cell Linker Mini Kit (Sigma-Aldrich, St. Louis, MO, USA) according to the manufacturer’s protocol. HUVECs (5 × 10^4^ per well) were seeded on a confocal dish overnight, followed by stimulation with PKH26-labeled EVs for 24 h. We fixed HUVEC with 4% paraformaldehyde for 10 min before staining the cell nuclei with DAPI. Images were taken with a confocal microscope (Zeiss LSM 800, Germany).

### CCK-8 cell proliferation assay

Proliferation of HUVECs treated with 5 μg/ml DF-EVs was detected using a CCK-8 kit (APExBIO, Houston, TX, USA) according to the manufacturer's instructions. Analysis was carried out in triplicate for three separate experiments.

### Transwell migration assay

The vertical migration abilities of HUVECs were assessed using a transwell migration assay. Briefly, equal numbers of HUVECs treated under different conditions and suspended in serum-free medium were seeded into transwell inserts (Transwell-3421, Corning, NY, USA), which were placed in 24-well plates containing medium supplemented with 10% serum. The stained cells were counted from five randomly fields per chamber.

### Endothelial cell tube formation

HUVECs treated with different agents were dissociated and seeded in angiogenesis μ-slides (ibidi, Martin Reid, Germany) precoated with basement membrane matrix (10 μl/well, Corning, Middlesex county, MA, USA) at a density of 1.0 × 10^4^ cells/well. Four to six hours later, images of capillary-like tube structures were acquired by light microscopy. The number and length of intact tubes per field were analyzed using ImageJ.

### MiRNA mimics and inhibitor transfection

MiRNA mimics and scrambled sequences (N.C.) were designed in Hongxun (Suzhou, China). After reaching 40–50% confluence, HUVECs were transiently transfected with miRNA mimics or N.C. (50 μM) by Lipofectamine 3000 (Invitrogen, Carlsbad, CA, USA). Cells were cultured for 48 h before harvesting for further experiments.

### qRT-PCR analysis

Total cellular RNA was isolated using an RNA-Quick Purification Kit (Yishan Biotechnology, Shanghai, China) according to the manufacturer’s instructions. Exosomal RNA was isolated according to the established protocol with TRIzol (Invitrogen, Carlsbad, CA, USA). qRT-PCR was proceeded on LightCycler 480 SYBR Green Master (Roche Diagnostics, Mannheim, Germany). β-actin and U6 were used as internal RNA standards for mRNA and miRNA respectively.

### MiRNA microarray analysis

MiRNA microarray analysis was performed by KangChen Biotech (Shanghai, China). The microarray was performed at platform GPL20712 based on miRbase v21, and the design ID is Agilent-070156 Human miRNA. MiRNA differentially expressing between groups with a fold change of ≥2.0 or ≤0.5, *P* < 0.01 were screen out. The miRNAs targeting VEGFA expression were predicted by FunRich software version 3.1.3, and images were drawn by Cytoscape software version 3.7.0. The 3′-UTR sequences of VEGFA were chemically synthesized, containing wild-type (WT) or mutated (MUT) binding sites for miR-195-5p or miR-205-5p distinctively. These sequences were inserted into the pGL3-luciferase reporter vector (Promega). Human embryonic kidney 293T cells were co-transfected with different reporter vector mentioned above by the Lipofectamine 3000 (Invitrogen). Luciferase activity of the cell lysates collected after 48 h was detected with a Dual-Luciferase Reporter Assay Kit (Promega).

### Western blot analysis

To identify the expression level of cellular VEGFA in HUVECs treated with different agents, lysates from cultured HUVECs were collected and analyzed, and western blot analysis was performed as described previously [16]. The antibody source and dilution were as follows: mouse anti-VEGFA. β-actin was used for normalization.

### *In vivo* wound model

Sprague-Dawley male rats each with a weight about 200–250 g were purchased from the Laboratory Animal Center of Sun Yat-Sen University. A wound was made in the dorsal skin as previously reported [16]. Immediately after the wound was established, Control-EVs or DF-EVs (200 μg solved in PBS) were injected subcutaneously around the wound edges. Wound images were obtained on day 0, 4 and 7. Wound healing rate was calculated as the following formula: (initial area-final area)/initial area × 100%.

### Hematoxylin and eosin (HE) staining and immunohistochemistry

Wound samples on day 7 were collected for histological analyses. HE was performed on wound sample sections to analyze the epidermal and dermal thickness. Avidin–biotin complex method was applied to immunohistochemistry [16]. New blood vessel formation of the wound tissues was detected by CD31 antibody (ab182981, Abcam). Horseradish peroxidase (HRP)-labeled second antibody revealed the primary antibody binding condition, which was visualized by streptavidin-biotin complex. Five high-power fields per section were obtained to conduct the Immunoreactive score (IRS) standard as described [16].

### Statistical analysis

Data were presented as mean ± standard deviation (SD). Student’s *t*-test or one-way ANOVA were used to analyze the differences among groups. Statistical analysis was performed with GraphPad Prism software version 5.0 and SPSS software version 23, and *p* < 0.05 was considered to be statistically significant.

## Supplementary Materials

Supplementary Figures

Supplementary Table 1
