# Supplementary material for: MiR-195-5p and miR-205-5p in extracellular vesicles isolated from diabetic foot ulcer wound fluid decrease angiogenesis by inhibiting VEGFA expression
Source: Aging (Albany NY). 2021 Aug 9;13(15):19805–21. doi: 10.18632/aging.203393 (PMC8386552; doi:10.18632/aging.203393)
Supplement: Supplementary Figures [file aging-13-203393-s001.pdf]

SUPPLEMENTARY FIGURES

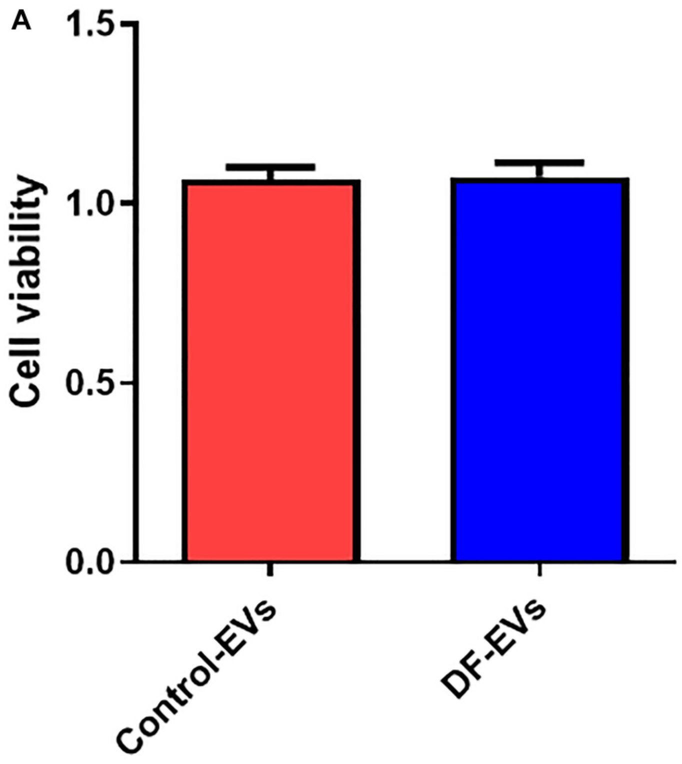

Supplementary Figure 1. (**A**) Cell viability of HUVECs treated with DF-EVs was detected by CCK-8. At least three replicates of each experiment were performed.

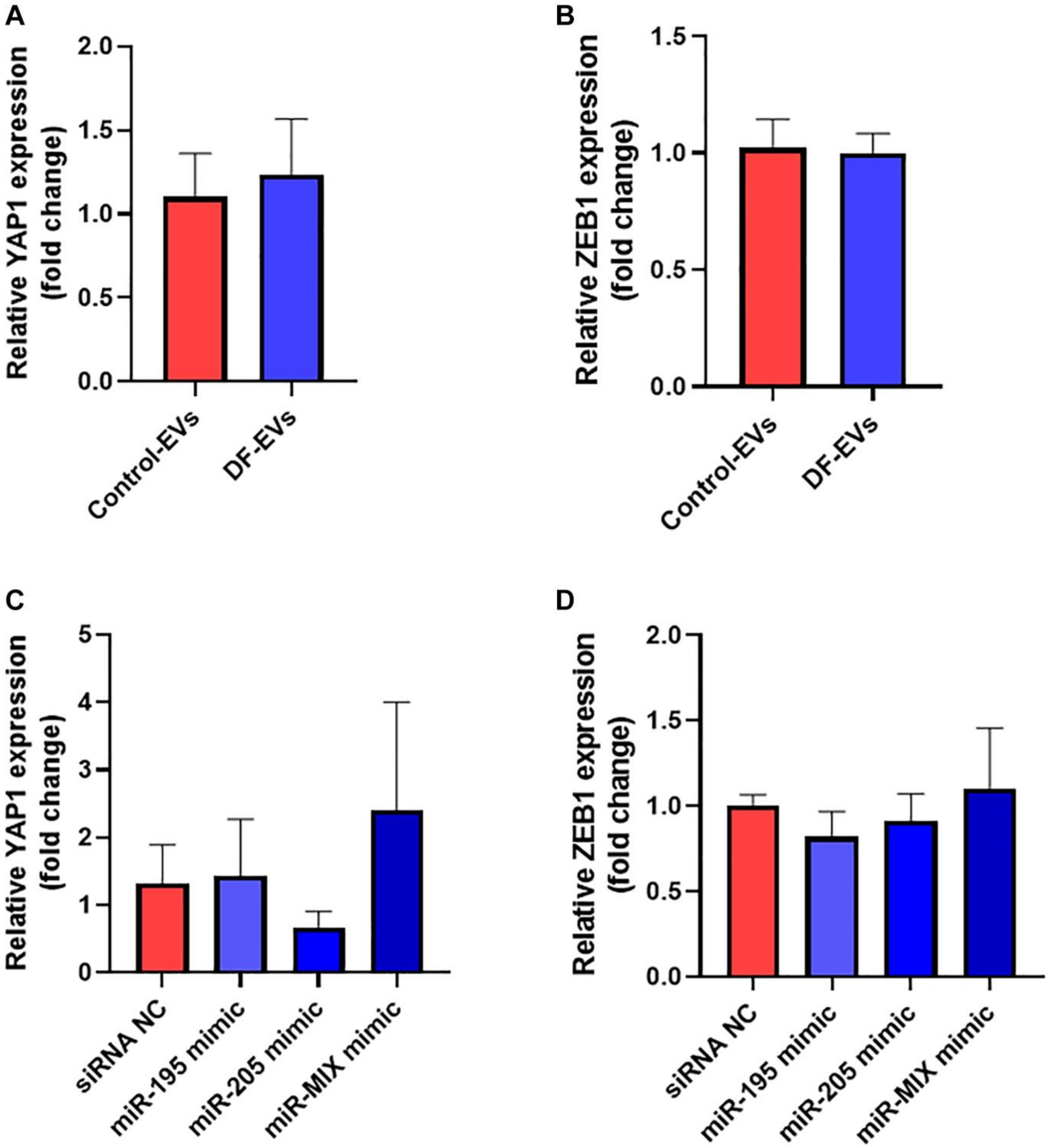

Supplementary Figure 2. Effects of DF-EVs, miR-195-5p and miR-205-5p on YAP1 and ZEB1. (**A**, **B**) Expression levels of YAP1 and ZEB1 in HUVECs treated with DF-EVs and Control-EVs was detected by qRT-PCR separately. (**C**, **D**) Expression levels of YAP1 and ZEB1 in HUVECs overexpressing either or both of miR-195-5p and miR-205-5p mimics was detected by qRT-PCR separately. At least three replicates of each experiment were performed.
