# Supplementary material for: MiR-195-5p and miR-205-5p in extracellular vesicles isolated from diabetic foot ulcer wound fluid decrease angiogenesis by inhibiting VEGFA expression
Source: Aging (Albany NY). 2021 Aug 9;13(15):19805–21. doi: 10.18632/aging.203393 (PMC8386552; doi:10.18632/aging.203393)
Supplement: Supplementary Table 1 [file aging-13-203393-s002.pdf]

SUPPLEMENTARY TABLE

Supplementary Table 1. Demographic and clinical characteristics of patients.

	DF	DM	Control	*P*
N (%)	21 (39.62)	14 (26.42)	18 (33.96)	
Age	65.19 ± 15.99	60.21 ± 9.72	61.54 ± 6.96	0.473
FBG (mmol/l)	7.49 ± 3.37*	8.50 ± 4.53**	5.37 ± 0.45	0.020
HbA1c (%)	8.10 ± 2.41**	9.47 ± 3.14***	5.56 ± 0.31	<0.0001
Cr (umol/l)	165.90 ± 115.65***	115.07 ± 71.88*	46.72 ± 36.99	<0.0001
TG (mmol/l)	1.35 ± 0.76	2.47 ± 3.45	1.33 ± 0.72	0.166
TC (mmol/l)	3.57 ± 0.79**	4.64 ± 1.59**	6.20 ± 1.27	<0.0001
LDL-C (mmol/l)	2.31 ± 0.58**†	2.99 ± 1.07	3.24 ± 0.94	0.004
HDL-C (mmol/l)	0.79 ± 0.22***	0.89 ± 0.25***	1.69 ± 0.43	<0.0001

Date are presented as mean ± SD; $^{*}P < 0.05$ vs. control, $^{**}P < 0.01$ vs. control, $^{***}P < 0.001$ vs. control, $^{\dagger}P < 0.05$ vs. DM.
